# Industrial Scale Manufacturing and Downstream Processing of PLGA-Based Nanomedicines Suitable for Fully Continuous Operation

**DOI:** 10.3390/pharmaceutics14020276

**Published:** 2022-01-25

**Authors:** Maria Camilla Operti, Alexander Bernhardt, Vladimir Sincari, Eliezer Jager, Silko Grimm, Andrea Engel, Martin Hruby, Carl Gustav Figdor, Oya Tagit

**Affiliations:** 1 Department of Tumor Immunology, Radboud Institute for Molecular Life Sciences, Radboud University Medical Center, 6500 HB Nijmegen, The Netherlands; maria-camilla.operti@evonik.com (M.C.O.); carl.Figdor@radboudumc.nl (C.G.F.); 2 Evonik Operations GmbH, Research Development & Innovation, 64293 Darmstadt, Germany; alexander.bernhardt@evonik.com (A.B.); silko.grimm@evonik.com (S.G.); 3 Institute of Macromolecular Chemistry CAS, Heyrovsky Square 2, 162 06 Prague, Czech Republic; sincarivl@gmail.com (V.S.); jagereliezer@gmail.com (E.J.); mhruby@centrum.cz (M.H.); 4 Evonik Corporation, Birmingham Laboratories, Birmingham, AL 35211, USA; andrea.engel@evonik.com

**Keywords:** PLGA, poly(lactic-co-glycolic acid), nanomedicine, nanoparticles, scale-up manufacturing, clinical translation, inline sonication, tangential flow filtration, lyophilization, downstream processing

## Abstract

Despite the efficacy and potential therapeutic benefits that poly(lactic-co-glycolic acid) (PLGA) nanomedicine formulations can offer, challenges related to large-scale processing hamper their clinical and commercial development. Major hurdles for the launch of a polymeric nanocarrier product on the market are batch-to-batch variations and lack of product consistency in scale-up manufacturing. Therefore, a scalable and robust manufacturing technique that allows for the transfer of nanomedicine production from the benchtop to an industrial scale is highly desirable. Downstream processes for purification, concentration, and storage of the nanomedicine formulations are equally indispensable. Here, we develop an inline sonication process for the production of polymeric PLGA nanomedicines at the industrial scale. The process and formulation parameters are optimized to obtain PLGA nanoparticles with a mean diameter of 150 ± 50 nm and a small polydispersity index (PDI < 0.2). Downstream processes based on tangential flow filtration (TFF) technology and lyophilization for the washing, concentration, and storage of formulations are also established and discussed. Using the developed manufacturing and downstream processing technologies, production of two PLGA nanoformulations encasing ritonavir and celecoxib was achieved at 84 g/h rate. As a measure of actual drug content, encapsulation efficiencies of 49.5 ± 3.2% and 80.3 ± 0.9% were achieved for ritonavir and celecoxib, respectively. When operated in-series, inline sonication and TFF can be adapted for fully continuous, industrial-scale processing of PLGA-based nanomedicines.

## 1. Introduction

Polymeric nanoparticles composed of biodegradable and biocompatible polymers such as poly(lactic-co-glycolic acid) (PLGA) display a promising future for various biomedical and pharmaceutical applications. However, the development of parenteral nanoparticle formulations for clinical and commercial use often faces the challenges of process scaling in a sterile or aseptic environment that complies with Good Manufacturing Practices (GMP) [1]. The main requirements for clinical and commercial development of nanoparticle formulations are high therapeutic efficacy and safety as well as production scalability and process robustness, which are closely connected with the material characteristics and applied production technology [2,3,4]. Therefore, the ability to establish large-scale processes for GMP-compliant production is indispensable for translating nanoparticle formulations from the bench to the bedside [1,5].

Emulsion-based methods are among the most exploited approaches for the preparation of PLGA nanoparticles, in which organic solvents are removed by evaporation or extraction. Equipment used currently used for the crucial homogenization step comprise probe sonicators, high-shear mixers, high-pressure homogenizers, and microfluidic systems [1,6]. In particular, emulsification by direct sonication with a transducer probe immersed in the processed medium is a highly common laboratory-scale approach due to the ease of operation that allows for rapid formulation screening [6,7]. However, direct contact of the drug product with the metal probe can lead to cross-contamination (e.g., with heavy metals), and the harsh homogenization shear and high temperature created during the cavitation phenomena can affect sensitive APIs and even result in the degradation of the polymeric carrier chains [1,7,8,9]. Besides, increased throughput can lead to changes in the formulation properties, making the technology only suitable for small batch preparations [1,6,7]. Sonication can also be applied indirectly via distributing the energy to the sample container itself, preventing the risk of sample contamination. The process can be entirely constructed using disposable materials (e.g., tubing and vessels) and operated inline in fully enclosed containers, which makes the process scalable and suitable for aseptic manufacturing [7,10].

During scale-up manufacturing, downstream processes are also necessary to isolate materials in the desired form and purity. Based on the preparation method, various impurities that can be toxic are present in the final product [11]. These impurities may include organic processing solvents such as dichloromethane (DCM) and dimethyl sulfoxide (DMSO), emulsifiers and stabilizers, monomeric residues, free unbound payloads, and salts. Such substances can lead to potential biological intolerance and alter the physicochemical characteristics of the nanoformulations. Therefore, an effectual cleaning strategy of nanoformulations is essential for controlling the quality and characteristics of nanomedicine products [11]. Lab-scale purification of nanoparticles is usually achieved by centrifugation, extraction, or filtration-based techniques. Most of these traditional techniques are time-consuming and often lack reproducibility, which can make these processes relatively inefficient [1,11]. Moreover, the requirement for manual handling can lead to more laborious processes when scaled up and is difficult to perform in an aseptic environment. Tangential flow filtration (TFF) is an alternative purification method that involves fluid flow along the surface of a membrane rather than passing through a filter. Scale-up of TFF processes is possible and is commonly considered straightforward since membrane cartridges are linearly scalable. Moreover, TFF can be operated in two modes within downstream unit operations: batch and single pass. The former involves recirculation of the processed solution over the membrane surface multiple times, while the latter operates in a single step, enabling a completely continuous operation [12]. As a continuous and gentle process that can significantly reduce membrane clogging compared to other traditional filtration methods, TFF is suitable for downstream processing of nanoparticles at the industrial level [1].

In this study, we developed PLGA nanoparticle manufacturing and downstream processes suitable for industrial scale-up. A scalable inline indirect sonication technique similar to that previously reported by Freitas et al. [10] was herein adapted, developed, and enhanced for the preparation of PLGA nanoparticles of approximately 150 ± 50 nm diameter with a polydispersity index (PDI) < 0.2. The particle size, PDI, zeta potential, and PLGA polymer chain integrity of different formulations were studied. TFF was tested and evaluated for different diafiltration volumes to determine the amount of impurities in the final drug product before storage. The need to use a cryoprotective agent during freeze-drying of the drug product was demonstrated. The results were evaluated following a statistical analysis to determine the significance of the difference among the compared groups. Ultimately, the inline sonication technique was scaled up for the production of nanomedicines containing two small drug molecules: ritonavir and celecoxib. The two model drugs used in this work have already been employed in the literature for the production of PLGA-based nanoparticles to overcome problems such as low patient therapy adherence [13] and important side effects [14]. The main reasons for the choice of these substances for large-scale experiments are the low toxicity of the compounds that are to be used in large quantities and their common availability.

## 2. Experimental Section

### 2.1. Materials

RESOMER RG 502 H (PLGA) (lactide–glycolide mole ratio of 50:50, inherent viscosity 0.16–0.24 dL/g) is an in-house product of Evonik Nutrition & Care GmbH (Darmstadt, Germany). Dichloromethane (DCM) ≥ 99.5% and ethyl acetate (EtOAc) ≥ 99.5% were purchased from Avantor Performance Materials (Gliwice, Poland) and used without further purification. Dimethyl sulfoxide (DMSO) 99.9%, USP grade, was acquired from WAK-Chemie Medical GmbH (Steinbach, Germany), and polyvinyl alcohol (PVA) was procured from Sigma-Aldrich (St. Louis, MO, USA). Trehalose dihydrate, boric acid, and Lugol’s iodine solution were purchased from Carl Roth GmbH + Co. KG (Karlsruhe, Germany). Ritonavir ≥ 98% was acquired from Angene International Limited (Hong Kong, China) and celecoxib 99.92% was procured from Aarti Drugs Ltd. (Mumbai, IN, USA).

### 2.2. Nanoparticle Production

#### 2.2.1. Placebo Formulation Development

##### Probe Direct Sonication

To obtain placebo particles of approx. 150 ± 50 nm and a PDI < 0.2, 4.06 mL of an organic solution composed of 3.9 wt% PLGA, 74.4 wt% DCM, and 21.7 wt% DMSO (dispersed phase (DP)) and 12.18 mL of an aqueous phase containing 2 wt% PVA (continuous phase (CP)) were emulsified together using a UP200St Ultrasonic Lab Homogenizer (Hielscher Ultrasonic GmbH, Teltow, Germany) equipped with an S26d2D needle probe. The process parameters were set at 100% amplitude and 100% phase. The treatment duration was 2 min. During the process, the sample container was kept immersed in an ice bath to prevent the degradation of sensitive material due to the high temperature generated by ultrasound. Ultimately, 290 mL of MilliQ water was added to the dispersion. Process parameters are summarized in Table 1.

##### Inline Indirect Sonication

**Figure 1:**
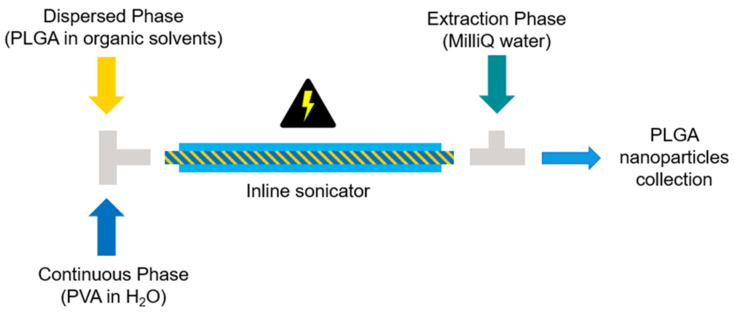
Scheme of the indirect inline sonication process setup for the manufacturing of PLGA nanoparticles. PLGA: poly(lactic-co-glycolic acid); PVA: polyvinyl alcohol.

To produce particles in an inline fashion, a GDmini2 Ultrasonic Inline Micro-Reactor (Hielscher Ultrasonic GmbH, Teltow, Germany) was used. This inline indirect sonotrode, consisting of a resonating stainless-steel jacket around a borosilicate glass tube, was connected to a pressurized coolant that transmitted the vibrations to the glass cannula during the process. Briefly, the same DP and CP solutions described above were prepared and filled inside two separate 60 mL BD Plastipak Luer-Lock single-use syringes and assembled on two Nexus 6000 syringe pumps (Chemyx Inc., Stafford, TX, USA). By connecting the two syringes to a T-junction, the DP and the CP were passed through the GDmini2 Ultrasonic Inline Micro-Reactor (Hielscher Ultrasonic GmbH, Teltow, Germany). DP:CP flow rate ratio was kept 1:3 and the total flow rate (TFR) was set at 2 mL/min. The pressurized coolant surrounding the glass cannula was maintained at a temperature of 10 °C through a cooling circulator (Ministat cc, Peter Huber Kältemaschinenbau AG., Offenburg, Germany) to avoid damage of sensitive raw materials during ultrasound treatment. Both the amplitude and phase of the indirect sonication process were kept at 100%. Prior to collection, another T-junction was coupled to the sonicator outlet, and MilliQ water (extraction phase (EP)) was pumped in through an ISCO pump (Teledyne ISCO, Lincoln, NE, USA) at 36 mL/min. Table 2 summarizes the process parameters applied. A schematic illustration of the process is depicted in Figure 1.

#### 2.2.2. Scale-Up of Inline Sonication

For the dispersed phase, 5, 10, and 20 wt% solutions of PLGA in EtOAc were prepared, mixed with DMSO, and processed at 8, 16, and 32 mL/min of TFR (Table 3, exp. 3–7). After having established the parameters to apply for the scale-up, 4 g of a 5 wt% solution of ritonavir or celecoxib in DMSO was added to 9 g of the 20 wt% solution of PLGA in EtOAc. The applied TFR of the DP/CP joint stream was set at 32 mL/min, and the resulting suspension was diluted with MilliQ water pumped at a rate of 240 mL/min by the ISCO pump (Table 3, exp. 8–9). After 1 h of stirring, the samples were purified and lyophilized. The final theoretical yield was calculated based on the composition and flow rate of the DP as 84 g/h. The obtained total volume containing the polymer and the API was divided by the flow rate of the DP to find the time necessary to process the materials. Finally, the grams of PLGA and APIs together were divided by the time to obtain the quantity of material processed per unit of time.

The scalability of the probe batch technique was also investigated (Table 4, exp. 10). Twelve milliliters of CP was added to 4 mL of the DP solution containing 20 wt% of PLGA in EtOAc mixed with DMSO, and the sample was sonicated for 30 s at 100% amplitude using an S26d2D needle probe. The final suspension was diluted with 81 mL of MilliQ water and stirred for 1 h prior to particle size characterization.

### 2.3. Analysis of Particle Size, Polydispersity Index, and Zeta Potential

The mean particle size diameter (Z-Average) and the polydispersity index (PDI) were determined via dynamic light scattering using a Zetasizer Nano ZS (Malvern Panalytical, Malvern, UK). Three measurements at 25 °C with a 173° scattering angle were conducted on each sample, which was previously diluted in sterile filtrated MilliQ water (0.2 µm). The surface charge of the nanoparticles was investigated by zeta potential measurement at 25 °C with the same instrument using the Smoluchowski equation. The two measurements together call for approx. 8 min of analysis time.

### 2.4. Downstream Processes

#### 2.4.1. Purification

The formulations were purified via tangential flow filtration (TFF) technique employing a KrosFlo KR2i TFF System (Repligen). A Spectrum hollow fiber filter of the MidiKros module family based on modified polyethersulfone (mPES) material was chosen with a molecular weight cut-off (MWCO) of 750 kDa, fiber ID of 0.5 mm, and 20 cm effective length (D02-E750-05-N). Various diafiltration volumes (DVs) (3, 5, and 8) were investigated, and the ability to remove the excess of stabilizer and residual organic solvents was evaluated for each DV.

#### 2.4.2. Analysis of Impurities

##### Proton Nuclear Magnetic Resonance (^1^H-NMR)

^1^H-NMR spectroscopy method was chosen as the analytical method for DMSO and DCM quantification. Despite gas chromatography–mass spectrometry (GC-MS) being a more sensitive, accurate, and suitable method for regulatory purposes, it is time-consuming and expensive. Conversely, the NMR method is advantageous for routine analysis because it minimizes sample preparation and has a fast measurement interval, saving time, money, and environmentally unfriendly organic solvents [15]. Although a disadvantage of NMR could be the higher detection limit compared to GC-MS, the amount of DCM and DMSO detected in our studies resulted satisfactory for the specific purpose.

Briefly, 200 µL of deuterated water (D2O) was added to 400 µL of each sample. Spectra were recorded using a Bruker 600 MHz spectrometer. Calibration was obtained based on the peak integrals related to a known amount of pure solvents. This method was used before, showing reproducible results [16]. The quantification limits (LOQs) for 1H-NMR are in a range of 1–15 μg/mL according to the literature [17,18,19].

##### Colorimetric Assay

The amount of residual PVA was determined by a colorimetric method based on the formation of a colored complex between two adjacent hydroxyl groups of PVA and an iodine molecule [20,21]. Briefly, 500 µL of each sample suspension was treated with 200 µL of 0.5 M sodium hydroxide solution for 15 min in a bath sonicator (BANDELIN electronic GmbH & Co. KG., Berlin, Germany) at 60 °C. Afterward, samples were neutralized with 90 µL of 1 M hydrochloric acid. To each sample, 300 µL of a 0.65 M solution of boric acid and 50 µL of Lugol’s iodine solution (I2/KI) were added. Finally, after 15 min of incubation at 700 rpm, the absorbance of the samples was measured by setting the absorbance of an Infinite M200 PRO (Tecan, Männedorf, Switzerland) at a wavelength of 689 nm. A standard plot of PVA was prepared using known PVA concentrations under identical conditions.

#### 2.4.3. Lyophilization

**Figure 2:**
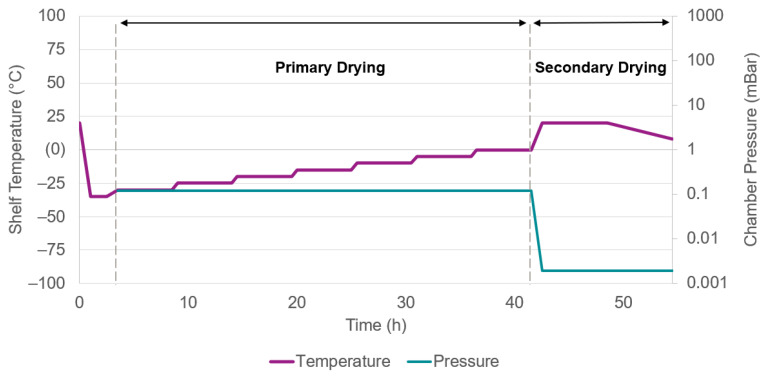
Freeze-drying protocol of the PLGA-based nanoparticles.

The purified suspensions were lyophilized using an Epsilon 2–6D LSCPlus freeze-dryer (Martin Christ Gefriertrocknungsanlagen GmbH, Osterode am Harz, Germany) in the presence of a cryoprotectant. A 40 wt% trehalose dihydrate stock solution was prepared and added to the colloidal suspensions to reach a 3 wt% final concentration. Figure 2 summarizes the experimental details of the freeze-drying protocol.

### 2.5. Molecular Weight Characterization

The molecular weight distribution (Mw, Mn, Mw/Mn) and inherent viscosity (η_inh_) of the processed polymer were determined by size exclusion chromatography (SEC) and viscometry methods. Experiments 1 and 2 were reproduced with the exception that the CP phase consisted of pure MilliQ water to avoid the interference of PVA with the PLGA chain measurements. Samples were lyophilized in the absence of cryoprotectant before the analysis.

#### 2.5.1. Gel Permeation Chromatography (GPC)

GPC analysis was performed on a setup equipped with VD400 Viscometer detector, RI200 RI detector, A5250 autosampler (Watrex Praha s.r.o., Prague, Czech Republic), and S 3210 UV/VIS detector (SYKAM GmbH, Eresing, Germany), using PLgel 5 µm 100 Å (Agilent Technologies, SC, USA) and DeltaGel Mixed-B (Watrex Praha s.r.o., Prague, Czech Republic) columns. Briefly, 5 mg of each sample was dissolved in 1 mL of a mixture consisting of chloroform:trimethylamine:isopropanol in 94:4:2 vol% composition. The same mixture was also employed as the mobile phase with a flow rate of 1 mL/min. The weight-average molecular weight (Mw), number-average molecular weight (Mn), and polydispersity (Đ) values were calculated using poly(methylmethacrylate) as standards (PSS Polymer Standards Service GmbH, Mainz, Germany).

#### 2.5.2. Inherent Viscosity (η_inh_)

One hundred milligrams of PLGA was dissolved in 100 mL chloroform and stirred for a minimum of 6 h to obtain a polymer solution. Before starting the measurements, an Ubbelohde viscometer was filled and equilibrated to 25 °C for 5 min. The flux time of the polymer solution between two marks was recorded, and the flux time of pure chloroform was measured as a reference. Two measurements were performed for each sample, and the η_inh_ was calculated using Equation (1):

η_inh_ = ln η_rel_/c (1)

where η_rel_ is the relative viscosity, which is the flux time ratio of the polymer solution to pure chloroform, and c is the polymer concentration having the unit of grams per deciliter.

### 2.6. API Content Analysis

For the API content analysis, high-performance liquid chromatography (HPLC) composed of a DIONEX UltiMate 3000 Pump and Diode Array Detector (UV-Vis) (Thermo Fisher Scientific, Waltham, MA, USA) was employed.

Drug encapsulation efficiency (EE) was calculated using Equation (2):

(2) $$\mathrm{EE}\;\left(\%\right)=\frac{\mathrm{Weight}\;\mathrm{of}\;\mathrm{drug}\;\mathrm{found}\;\mathrm{in}\;\mathrm{the}\;\mathrm{nanoparticles}}{\mathrm{Weight}\;\mathrm{of}\;\mathrm{drug}\;\mathrm{initially}\;\mathrm{used}}\times 100$$

#### 2.6.1. Ritonavir

Ritonavir quantification was conducted using a Nucleosil 100-7 C18 (125 × 4.6 mm; 7 µm) column. Standard calibration curves were performed at a fixed wavelength of 215 nm. The column temperature was maintained at 22 °C. The mobile phase comprised 30 mM potassium dihydrogen phosphate with pH adjusted to 4.0 with orthophosphoric acid and acetonitrile (45:55 vol%) with a flow rate of 1.5 mL/min. Injection volume was 10 µL. Retention time was 4.2 min. DMSO was employed to prepare the standard calibration solutions and the nanoparticle samples.

#### 2.6.2. Celecoxib

Celecoxib was detected using a Nucleosil 100-7 C18 (125 × 4.6 mm; 7 µm) column. Eluents were monitored at a wavelength of 254 nm using a mixture (50:50:0.15 vol%) of acetonitrile, water, and triethylamine (TEA) with pH adjusted to 3.0 with orthophosphoric acid. Flow rate was kept at 1.8 mL/min and the column temperature was maintained at 40 °C. Injection volume was 5 µL and retention time was recorded at 4.1 min. Standard calibration and samples were formulated in DMSO.

### 2.7. Statistical Analysis

Student’s *t*-test (two-tailed distribution, homoscedastic) (*n* = 3) was used in the polymer chain length analysis to determine the significance of the difference (*p* < 0.05) in Mw and η_inh_ among the compared groups with regards to the unprocessed PLGA powder. It was also exploited to verify the significance of the difference in mean particle size values for downstream processing impact analysis.

## 3. Results and Discussion

### 3.1. Assessment of Process and Formulation Parameters for Lab-Scale Production

PLGA nanoformulations were developed at a laboratory scale using both probe sonication and inline sonication with the same dispersed phase (DP) composition (3.9 wt% PLGA, 74.4 wt% DCM, 21.7 wt% DMSO; Table 1 and Table 2). Chlorinated DPs for the emulsion-based preparation of nanosized PLGA formulations are commonly found in the scientific literature [1,22]. DMSO was added to the organic phase prior to sonication in order to mimic the exact conditions commonly used for the encapsulation of (bio)therapeutic payloads [23]. DMSO is a polar aprotic solvent with the ability to dissolve both polar and nonpolar compounds and is miscible with a wide range of organic solvents [24]. Addition of DMSO to the limitedly water-miscible DCM usually results in the formation of smaller emulsion droplets for a given set of process and formulation parameters due to lowering of the interfacial tension by DMSO [6,25,26], which is unlimitedly miscible with water [27,28]. Following the sonication process (probe sonication), an excess amount of MilliQ water was mixed with the crude emulsion to extract the organic solvents from the suspension to harden the particles, thereby accelerating the downstream processes. In view of a possible commercial and GMP-compliant manufacturing process, it is desirable to have a fast particle surface hardening process to decrease the waiting time of evaporation of a volatile solvent such as DCM. Depending on the water solubility of the organic solvent and its affinity for PLGA [29], the hardening process may take a variable amount of time. Based on the solubility of DCM in water (1.3 g/100 mL at 25 °C [30]), approx. 77 mL of water was added for each gram of DCM used for an efficient extraction.

The starting formulation tested for the probe sonication method was kept unchanged and processed at the inline sonicator. In the inline indirect sonication method, the sample flows continuously inside of a glass tube. The glass cannula is surrounded by a pressurized water coolant, which transmits the ultrasonic waves generated by the sonotrode by means of mechanical vibrations, allowing the formation of a fine emulsion. In this process, the glass tube is the only material in contact with the sample, rendering the procedure free from cross-contamination (e.g., metal shards from the probe, impurities derived from the surrounding environment) and potentially suitable for sterile processing [7]. The glass tube can be for single use or sterilized before usage for GMP-compliant processes.

During the process, flow stability was maintained through the use of syringe pumps, which can provide a constant fluid flow. Although syringe pumps are limited by the loading capacity of the syringes, they can be easily replaced by HPLC pumps to process larger volumes. In either case, pipes must conform to certain specifications with regard to compatibility with harsh solvents. In addition, it is important to note that an inline technology consisting of multiple tubes can generate a dead volume and some of the material can therefore be lost. In the setup described in this work, approximately 15 mL of dead volume was generated within the inline apparatus, which can be reduced by, for example, decreasing the length or diameter of the tubes in the system.

**Figure 3:**
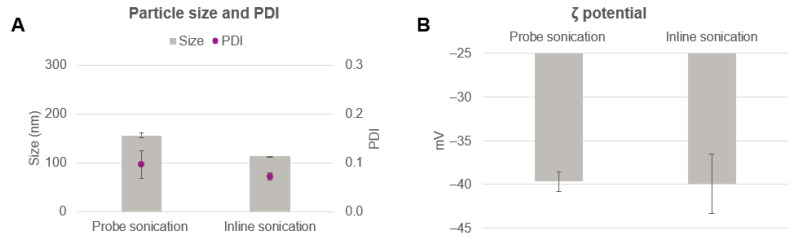
(**A**) Particle size and PDI and (**B**) zeta potential of placebo PLGA nanoparticles generated via probe direct and inline indirect sonication.

The placebo nanoparticle characteristics obtained for both sonication methods are shown in Figure 3. Figure 3A shows that a slightly smaller particle size compared to the classical probe method was obtained with the inline sonication method and highly monodisperse particles (PDI < 0.1) were generated for both preparations. The zeta potential value of PLGA nanoparticles obtained by the probe and inline sonication methods was around −40 mV (Figure 3B) due to the utilization of the same formulation parameters in terms of PLGA and PVA concentrations.

### 3.2. Downstream Process Development for Washing, Concentration, and Storage of PLGA Nanoparticles

**Figure 4:**
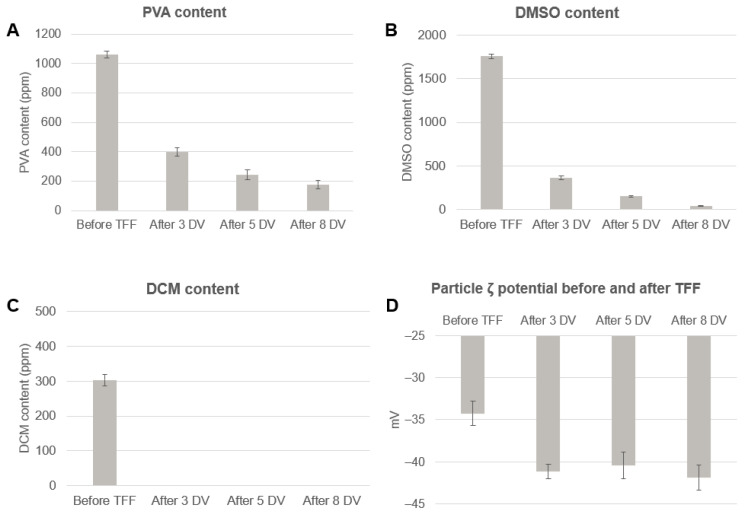
Downstream process evaluation. Reduction in (**A**) PVA, (**B**) DMSO, and (**C**) DCM content and (**D**) particle zeta potential tested before and after 3, 5, and 8 DVs.

**Figure 5:**
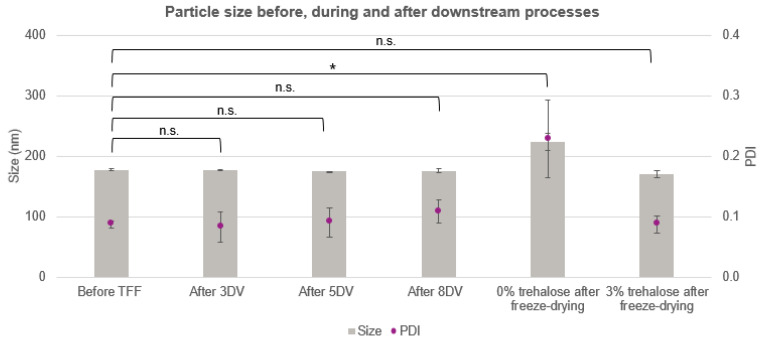
Size and size polydispersity of the nanoformulations before, during, and after the downstream processes. Significant, *p* < 0.05 (*); nonsignificant, *p* > 0.05 (n.s.).

The obtained diluted placebo suspension was first subjected to a concentration treatment corresponding to 6 times the initial volume, and then to three, five, and eight washing cycles (called diafiltration volumes (DVs)) using the TFF. The reason for choosing such DVs was dictated by the fact that typically, when washing samples by centrifugation, at least three wash cycles are used. In this work it was decided to start from a similar concept, i.e., to use three DVs and then scale up to five and eight. A single DV corresponds to the sample volume that is processed when the diafiltration starts. The washing liquid is added at the same rate as the discarded filtrate liquid is generated. When the volume of filtrate collected is equal to the initial sample volume, one DV is processed. The organic solvent content as well as the amount of stabilizer present in the suspensions were analyzed before and after each passage. The particle size and polydispersity were monitored during the process to ensure that particle properties were not affected by the TFF process. Furthermore, zeta potential was checked after each tested DV given its shielding effect on PLGA nanoparticle surface charge [21]. Figure 4 demonstrates the impact of diafiltration volumes on the PVA (Figure 3A), DMSO (Figure 4B), and DCM (Figure 4C) levels. While three diafiltration volumes were enough to completely eliminate DCM (Figure 4C), a significant drop in DMSO and PVA content after three diafiltration volumes was registered, and a gradual reduction after five and eight volumes was observed. Table 5 summarizes the reduction in DCM, DMSO, and PVA using TFF, showing that no traces of DCM were found in suspension, and that approx. 80% of DMSO and more than 60% of total PVA amount were removed already after three diafiltration volumes. The zeta potential measurements revealed negative values (Figure 4D), which are typically observed for uncapped end carboxylic groups of PLGA or hydrolyzed PLGA. However, these values were observed to be less negative for the nanoformulation not yet washed, thus containing a higher quantity of PVA, compared to those where PVA was substantially removed. In fact, as already observed by other groups [21,31], PVA presence is directly connected to the resulting zeta potential. Partially hydrolyzed PVA is an amphiphilic “multiblock” copolymer possessing poly(vinyl acetate) and poly(vinyl alcohol) blocks. This polymer structure is preferred because the hydrolysis rate of vinyl acetate monomeric units is strongly accelerated if the unit in the neighborhood is already hydrolyzed. Thus, once a unit in the poly(vinyl acetate) is hydrolyzed, it speeds up the hydrolysis of its neighbors. The hydrophobic part composed of poly(vinyl acetate) may be found associated with the polymer matrix of PLGA, while the hydrophilic poly(vinyl alcohol) can protrude from the surface of the nanoparticle [21,31]. Thereby, most of the PVA located close to the particle surface can shield the negative charges generated by PLGA [32]. Figure 4D highlights that three DVs are sufficient to remove the excess of PVA which greatly shielded the surface charge of the particles, leaving only the necessary surfactant that is attached to the surface and contributes to the overall stability of the nanoparticles. Finally, Figure 5 shows that the particle size and PDI remained unaffected by the flow filtration process, meaning that the process is well tolerated by the colloidal suspensions regardless of the number of diafiltrations performed. Based on these results, three DVs were chosen for washing the API-containing PLGA particles since the concentration of DCM and DMSO was reduced by more than approx. 80%, excess of PVA was removed revealing a more negative zeta potential of the particles, and size and PDI were not altered.

In order to prevent the nanoparticle agglomeration, flocculation, and premature release of APIs from the polymeric carrier, a lyophilization process was developed for nanoparticle storage. For these trials, the efficacy of a cryoprotectant (i.e., trehalose) to preserve the particle size and polydispersity of the final formulations was investigated. Trehalose is a nonreducing disaccharide consisting of two D-glucose monomers linked by an α,α–1,1 glycosidic bond [33]. It is found in bacteria, fungi, plants, and animals (e.g., tardigrades) that can survive extreme temperatures and withstand dehydration [33]. In the dried state, carbohydrates such as trehalose exert their protective effect by acting as a water substitute [34]. Besides, trehalose is an excipient already used in approved injectable products such as Herceptin (trastuzumab), Advate (octocog alfa), and Avastin (bevacizumab). As shown in Figure 5, when the lyophilization was performed without the addition of the cryoprotectant, the mean particle size increased above 200 nm and the PDI doubled in value. Conversely, the addition of trehalose in the suspension (in 3 wt% final concentration) prevented particles from agglomerating and preserved nanoparticle size and PDI.

### 3.3. Characterization of Polymer Chain Integrity after Sonication Processes

**Figure 6:**
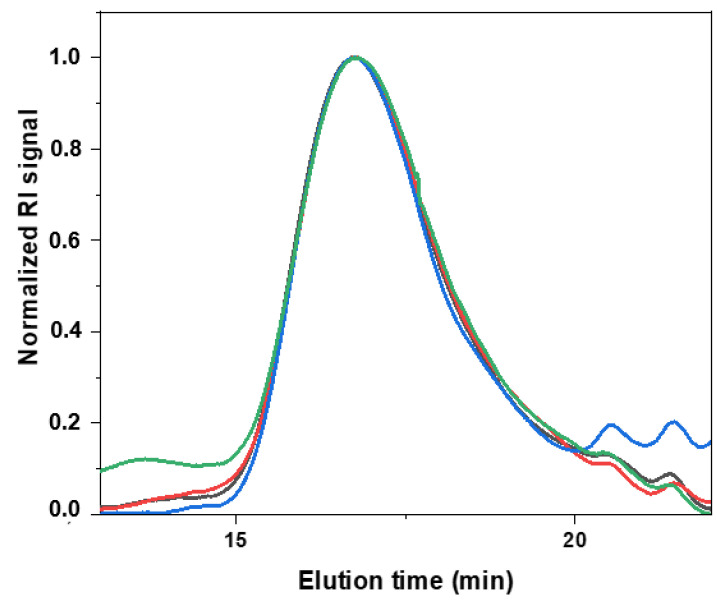
GPC spectra of the processed and untreated polymer under ultrasound. Data obtained for PLGA pure powder untreated (black), PLGA dissolved in the organic solvent (green), and PLGA treated under probe (red) and inline (blue) ultrasound technologies are presented.

Having obtained similar particles by both techniques, the methods were compared regarding their ability to keep the PLGA polymer chains intact as high shear forces exerted during sonication step can cause polymer chain rupture and degradation [35]. This is highly undesirable since it can both modify the structure of the carrier, which could result in altered phenomena (e.g., encapsulation efficiency and drug release), and generate reactive structures that could react with other species in suspension (e.g., payloads, stabilizers, etc.) [8]. Therefore, the PLGA chain lengths were analyzed using gel permeation chromatography (GPC) and viscometry before and after the ultrasound treatment in comparison to unprocessed PLGA powder. The normalized GPC spectra are shown in Figure 6, and the Table 6 and Table 7 summarize the results.

As sonication may cause a drop in molecular weight of polymers due to shear-induced chain breaks [8,9], the molecular weight of PLGA before and after processing was analyzed and compared. GPC analysis showed that the type of PLGA employed (RG 502 H) has a weight-average molecular weight Mw of approx. 19 kDa and an inherent viscosity of approx. 0.22 dL/g, which is in line with the product specifications reported in the certificate of analysis of RESOMER RG 502 H (0.16–0.24 dL/g). Although the drop in inherent viscosity results of PLGA treated with the inline sonication method showed a statistical significance at *p* ≤ 0.05, the difference is still only 6% of the original value, meaning that neither of the techniques affected the polymer chains during the manufacturing of particles. Negligibility of this difference and preservation of the original polymer properties is underlined by the fact that the original PLGA has a relatively wide distribution of molecular weights (PDI 1.59), so differences in molecular weight of ca. 4% are meaningless (also, within the experimental error of GPC).

### 3.4. Scale-Up of Formulation

**Figure 7:**
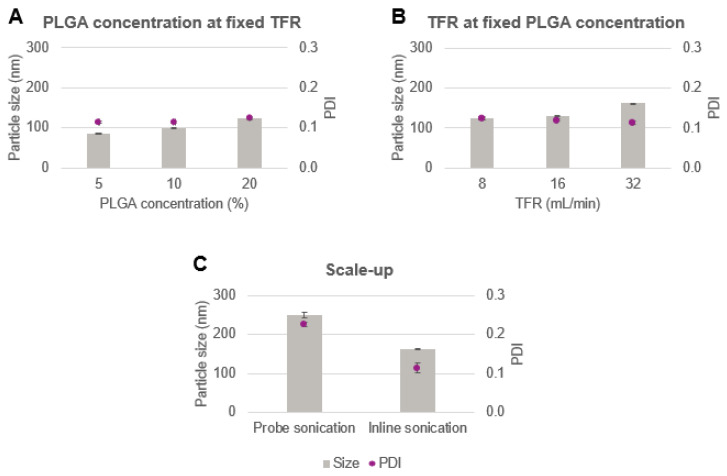
Scale-up of nanoformulations. (**A**) Size and PDI of the particles obtained at varied PLGA concentrations and fixed total flow rate of 8 mL/min. (**B**) Particle size and size distribution of placebo particles obtained applying different TFRs by using an initial PLGA concentration of 20 wt% in EtOAc. (**C**) Comparison of the particles obtained with the scaled-up indirect inline technology versus particles achieved at a higher processed volume and lower total sonication time of direct probe batch method.

The inline indirect sonication method was further investigated for PLGA-based nanomedicine scale-up manufacturing. First, the organic solvent used to prepare the polymer solution was switched from DCM to EtOAc due to the lower toxicity and higher water solubility of EtOAc compared to DCM. In fact, in accordance with the ICH guidelines [36], DCM is a class 2 solvent with a permitted daily exposure (PDE) equivalent to 6.0 mg/day. Conversely, EtOAc lies in class 3, meaning that the PDE corresponds to 50 mg/day [36]. Therefore, working with large amount of a less toxic solvent increases the safety of employees at work while reducing the complexity of safety protocols that must be prepared and heeded. Also, the higher degree of water solubility of EtOAc (8 g/100 mL versus 1.3 g/100 mL for DCM at 25 °C [30,37]) would require smaller volumes of water as the extraction phase and render the process easier to handle. Although therefore many substances should be reduced or directly replaced with greener ones, it is not always possible to choose the desired solvents because not all substances have the same solubility in various solvents. For example, super critical CO_2_ offers the advantages of being non-toxic and leaving no residue. On the other hand, the limited choice of soluble materials and the compatibility of organic solvents with the technical apparatus hinder its industrial scalability [1,38]. Therefore, in this case, during scale-up experiments, EtOAc was chosen over DCM because of its lower toxicity and unaltered ability to solubilize PLGA, while DMSO was selected for its ability to dissolve a wide variety of active ingredients. Thus, the DMSO/EtOAc mix was identified as optimal for most formulations to be manufactured. Nonetheless, it is important to note that, although notoriously toxic, the use of DCM in particle production is still widespread and has been reported for the production of formulations used in phase I clinical trials [39]. Keeping in mind the required specification of 150 ± 50 nm and PDI < 0.2, a set of particles was generated via indirect inline sonication technique with the scope of scaling-up the technology. The experiment parameters are reported in Table 3, exp. 3–7. The influence of polymer concentration and the TFR on the particle size and PDI were studied systematically. Figure 7A shows the variations of particle size as a function of PLGA concentration (5 wt%, 10 wt% and 20 wt%) obtained at a TFR of 8 mL/min. Given the greater water solubility of EtOAc compared to DCM, an extraction phase ratio of 3 (3 folds the amount of water required to solubilize EtOAc) was adopted, bringing the EP flow rate at 60 mL/min. This was done in order to dilute the particle suspension enough to be processed on the same day at the TFF, avoiding potential damage of the hollow fiber filter caused by the solvent. As expected, the use of EtOAc resulted in smaller particles than DCM due to the lower interfacial tension of the solvent with water, as previously observed in the case of DMSO addition. Also, as already experienced in one of our previous works [6], a gradual increase in particle size and PDI was noticed for higher concentrations of PLGA. While 5 wt% polymer resulted in particles with a mean diameter of approx. 85 nm, 125 nm particles were obtained with 20 wt% of polymer concentration with a PDI of approx. 0.1 for all formulations. As 10 wt% and 20 wt% PLGA were in the specification range, the higher PLGA concentration in EtOAc was chosen as the polymer concentration for the subsequent experiments due to the higher throughput of 19 g/h obtained with this polymer concentration. The influence of the TFR on the particle size and PDI is shown in Figure 7B. Accordingly, an increase in particle size was observed at higher TFR. This is due to shorter sonication time for the faster-flowing samples, leading to less homogenizing treatment time. 32 mL/min was found to result in particles still in the target size, therefore, it was chosen for the production of the loaded particles given the high throughput obtained from the high continuous flow (approx. 76 g/h).

As a comparison, the batch production method was also implemented in large scale (Table 4, exp. 10). Briefly, 20 wt% *w/w* PLGA in EtOAc was mixed with DMSO and sonicated for 0.5 min with PVA 2 wt%. Subsequently, the homogenized suspension was transferred to 81 mL of water used as the extraction phase. Figure 7C shows the particle size produced by this method. As it can be noted, the particle size as well as the PDI are out of the required specifications, and the values are considerably higher than those obtained with the inline method. This shows that inline sonication is easier to scale up and that, to allow for increased batch scale, a new process with a different probe would have to be entirely reevaluated.

#### Ritonavir and Celecoxib Nanoparticles

To further confirm the usefulness of the inline production method, ritonavir and celecoxib were chosen as model drugs and were encapsulated within PLGA nanoparticles. Ritonavir is an antiretroviral protease inhibitor API that is widely used in combination with other medications for the treatment of human immunodeficiency virus (HIV) infection, which causes the acquired immunodeficiency syndrome (AIDS) [13]. Celecoxib is a cyclo-oxygenase-2 (COX-2) selective inhibitor used in the treatment of pain and inflammation [14]. It is one of the most commonly prescribed COX-2 specific inhibitors since its use effectively reduces clinical gastrointestinal events in comparison to other nonsteroidal anti-inflammatory drugs (NSAIDs). Both of these APIs have already been investigated regarding their ability to be entrapped into PLGA nanoparticles in order to overcome problems such as low patient therapy adherence [13] and important side effects [14].

**Figure 8:**
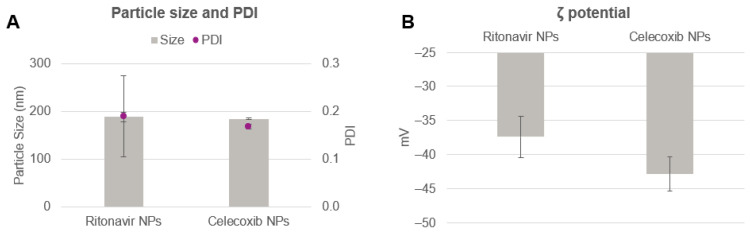
(**A**) Particle size and PDI and (**B**) zeta potential of ritonavir and celecoxib nanoparticles obtained with the inline scaled-up sonication method.

Since ritonavir and celecoxib are hydrophobic compounds, PLGA and API are dissolved together in the organic phase and then emulsified with the aqueous solution containing the surfactant. Otherwise, encapsulation of a hydrophilic API would require an initial formation of a water-in-oil emulsion, in which the API is in the aqueous phase and the polymer is in the organic phase. Next, the water-in-oil emulsion is mixed with a second aqueous solution containing the surfactant, creating a water-in-oil-in-water system [1]. The experimental conditions for large-scale manufacturing of ritonavir and celecoxib nanoparticles are shown in Table 3 (exp. 8–9). Both the nanoparticle types were produced achieving a total yield of 84 g/h, which corresponds to approx. 2 kg/day when run continuously. The characteristics of the nanoparticles are shown in Figure 8. The particle size achieved with this method was below 200 nm for both formulations. Ritonavir encapsulation led to particles of 188.5 ± 10.2 nm and PDI of 0.19 ± 0.09. Zeta potential was registered at −37.4 ± 3.0 mV, meaning that the particles were overall stable. Celecoxib resulted in particles of similar size and PDI of 184.7 ± 1.2 nm and 0.17 ± 0.01. Zeta potential was slightly more negative, being −42.8 ± 2.5 mV. Encapsulation efficiency of both the APIs was determined with HPLC. As shown in Table 8, the efficiency of ritonavir and celecoxib encapsulation was approx. 50% and 80%, respectively. Although ritonavir and celecoxib may possess some similar characteristics such as no formal charge and very poor water solubility, their functional molecular groups as well as molecular weights are substantially different [40,41]. Therefore, it is reasonable that each difference may decree a variation in the interaction of the API with the polymer matrix, resulting in a unique encapsulation capacity. Table 9 summarizes some of the most prominent physicochemical characteristics of the APIs.

## 4. Conclusions

Technologies that rely on inline continuous manufacturing processes allow the scaled-up production of nanomedicines without changing formulation specifications. Although inline processes may suffer from material loss due to dead volume in the tubes that necessarily increases the cost of upstream materials, this reliance makes such technologies attractive from a commercial and clinical development standpoint. In the present study, the indirect inline sonication method was found to be comparable to the direct probe method in terms of particle size, PDI, and zeta potential. Furthermore, it was confirmed to be safe as it does not alter the length of the polymer chains of PLGA during the sonication process. The inline sonication technology proved to be scalable, achieving a production yield of 84 g/h (approximately 2 kg/day) for PLGA nanoparticles containing ritonavir and celecoxib as modal APIs. Downstream processes have been developed and shown to be suitable to purify colloidal suspensions from impurities and residual organic solvents. Finally, due to the application of fully enclosed pipes and containers that can be easily sterilized or replaced, the inline indirect sonication method together with the TFF technique can potentially be considered for GMP and aseptic manufacturing processes. Overall, the developed manufacturing process proved to be suitable for the production of PLGA-based nanomedicine products on a clinical and commercial scale.

## Figures and Tables

**Table 1:** Process parameters for the classic probe sonication method.

Probe Sonication							
Exp. No.	DP Solution	DP Volume (mL)	CP Solution	CP Volume (mL)	Total Sonication Time (min)	EP Solution	EP Volume (mL)
1	3.9 wt% PLGA, 74.4 wt% DCM, 21.7 wt% DMSO	4.06	2 wt% PVA	12.18	2	MilliQ water	290

**Table 2:** Process parameters for the inline sonication method.

Inline Sonication							
Exp. No.	DP Solution	DP Flowrate (mL/min)	CP Solution	CP Flowrate (mL/min)	EP Solution	EP Flowrate (mL/min)	Throughput g/h
2	3.9 wt% PLGA, 74.4 wt% DCM, 21.7 wt% DMSO	0.5	2 wt% PVA	1.5	MilliQ water	36	1.6

**Table 3:** Process parameters evaluated for the inline sonication method scale-up.

Inline Sonication								
Exp. No.	API	DP Solution	DP Flowrate (mL/min)	CP Solution	CP Flowrate (mL/min)	EP Solution	EP Flowrate (mL/min)	Throughput (g/h)
3	-	4.5 wt% PLGA, 85.9 wt% EtOAc, 9.6 wt% DMSO	2	2 wt% PVA	6	MilliQ water	60	5
4	-	8.3 wt% PLGA, 74.3 wt% EtOAc, 5.7 wt% DMSO	2	2 wt% PVA	6	MilliQ water	60	10
5	-	14.1 wt% PLGA, 56.2 wt% EtOAc, 29.7 wt% DMSO	2	2 wt% PVA	6	MilliQ water	60	19
6	-	14.1 wt% PLGA, 56.2 wt% EtOAc, 29.7 wt% DMSO	4	2 wt% PVA	12	MilliQ water	120	38
7	Placebo	14.1 wt% PLGA, 56.2 wt% EtOAc, 29.7% DMSO	8	2 wt% PVA	24	MilliQ water	240	76
8	Ritonavir	13.9 wt% PLGA, 55.4 wt% EtOAc, 1.5 wt% ritonavir, 29.2 wt% DMSO	8	2 wt% PVA	24	MilliQ water	240	84
9	Celecoxib	13.9 wt% PLGA, 55.4 wt% EtOAc, 1.5 wt% ritonavir, 29.2 wt% DMSO	8	2 wt% PVA	24	MilliQ water	240	84

**Table 4:** Process parameters evaluated for the classic probe sonication method scale-up.

Probe Sonication								
Exp. No.	API	DP Solution	DP Volume (mL)	CP Solution	CP Volume (mL)	Total Sonication Time (min)	EP Solution	EP Volume (mL)
10	Placebo	14.1 wt% PLGA, 56.2 wt% EtOAc, 29.7 wt% DMSO	4	2 wt% PVA	12	0.5	MilliQ water	81.23

**Table 5:** Impact of different TFF diafiltration volumes in reducing PVA, DMSO, and DCM content.

Diafiltration Volumes	PVA Reduction (%)	DMSO Reduction (%)	DCM Reduction (%)
3	62.6 ± 3.3	79.4 ± 1.4	100.0
5	77.1 ± 3.7	91.4 ± 0.7	100.0
8	83.3 ± 2.4	97.5 ± 0.2	100.0

**Table 6:** Weight (Mw) and number (Mn) averaged molecular weight and PDI (Mw/Mn) of PLGA polymer treated and untreated via ultrasound. Significance of the difference (*p* < 0.05) among the compared groups was determined with regards to the unprocessed PLGA powder.

Gel Permeation Chromatography				
Sample	M_w_ (kDa) ± SD	M_n_ (kDa) ± SD	PDI	p Value (M_w_)
Untreated PLGA	18.77 ± 0.51	11.67 ± 0.51	1.59	
PLGA dissolved in DCM, untreated	19.52 ± 0.45	14.23 ± 0.99	1.35	0.13
PLGA treated with probe sonication	18.72 ± 0.56	12.41 ± 1.31	1.50	0.92
PLGA treated with inline sonication	18.80 ± 1.00	12.57 ± 1.43	1.50	0.96

**Table 7:** η_inh_ of the PLGA polymer treated and untreated via ultrasound. Significance of the difference (*p* < 0.05) among the compared groups was determined with regards to the unprocessed PLGA powder.

Viscometry		
Sample	η_inh_ (dl/g) ± SD	*p* Value
Untreated PLGA powder	0.219 ± 0.008	
PLGA dissolved in DCM, untreated	0.213 ± 0.001	0.24
PLGA treated with probe sonication	0.216 ± 0.004	0.57
PLGA treated with inline sonication	0.206 ± 0.001	0.05

**Table 8:** Ritonavir and celecoxib nanoparticle encapsulation efficiency and relative drug load obtained via the scaled-up indirect inline continuous method.

Drug Content	Ritonavir PLGA Nanoparticles	Celecoxib PLGA Nanoparticles
EE (%)	49.5 ± 3.2	80.3 ± 0.9
Drug load (mg/g)	4.95 ± 0.32	8.03 ± 0.09

**Table 9:** Ritonavir and celecoxib physicochemical characteristics. Data obtained consulting PubChem and DrugBank databases.

Physicochemical Characteristics	Ritonavir	Celecoxib
API Type	Small molecule	Small molecule
Mw	720.9	381.4
Log P	3.9	3.53
Hydrogen Bond Donors	4	1
Hydrogen Bond Acceptors	9	7
Formal Charge	0	0
Water Solubility (mg/L, 25 °C)	1.1 × 10^−4^	4.3

## Data Availability

Not applicable.
